# Self-Castration

**Published:** 1853-04

**Authors:** 


*Self-Castration.* From the Report of Prof. Gross to the Kentucky Medical Society.—The folllowing interesting case of self-castration has been communicated to me, through Dr. Frazee, of this city, by Dr. Samuel R. Sharpe, of Maysville:

W. O. N., aged twenty years, of a lymphatic temperament, light complexion, six feet high, and weighing one hundred and seventy-five pounds; a teamster and drover by occupation, came to Maysville in the autumn of the year 1849, having emasculated himself only the day previous. Calm, clear and collected upon every subject except that of his sexual organs, which he said had caused him more trouble than all the rest of his system put together, he viewed the act of mutilation in all its bearings and aspects with the utmost indifference, declaring that it was far better to part with the offending members than to be a slave to his passions. Looking upon the matter in this philosophical light, he had not hesitated to perform the operation, and now that it is over both his feelings and his conscience fully approved the act. He had no regrets, and he was satisfied that his future would be far more happy than his past life. This feeling of self-approbation continued for more than a year, but of late his conduct has been a source of deep chagrin and mortification to him.

The operation was performed in the woods, by himself, with a razor, at least half a mile from any dwelling, about five o'clock in the afternoon. There was but little external hemorrhage, but the blood collected in the scrotum, distending it to the size of a cocoa-nut. To guard against bleeding, he scraped the spermatic vessels, as he had often scraped those of a pig. The first testicle he removed, he said, in a very bungling manner, as his incisions were all too light; but the other was taken away very quickly and neatly. Hoisting his umbrella, he coiled himself up under it, and in this condition remained until the next morning, passing a calm and quiet night, not even thinking of the women. It is worthy of remark that this man labored under gonorrhea at the time of the operation, and that the removal of his testes completely cured this disease without the aid of local or constitutional treatment. The reporter of the case facetiously asks, what do you think of such a remedy for clap? Do you think it would become popular?

The treatment of the case after it fell into the hands of Dr. Sharpe was very simple. Poultices of cinchona and charcoal were used until the coagula were digested off, when they were replaced with cloths wet with warm water. A mild vegetable tonic was administered, and a nourishing diet enjoined. The patient left Maysville in about a month. Dr. Sharpe states that he occasionally sees this man, and that he can observe a sensible change in his hair and beard, both becoming thinner and softer.

His voice is unaltered, and there is no increase of flesh or fat. His industry and power of endurance are about the same as before the operation. He still retains some propensity for women, and declares that he has indulged in sexual intercourse with feeling of a pleasurable kind.

